# Polyelectrolyte Complex Dry Powder Formulations of Tobramycin with Hyaluronic Acid and Sodium Hyaluronate for Inhalation Therapy in Cystic Fibrosis-Associated Infections

**DOI:** 10.3390/antibiotics14020169

**Published:** 2025-02-08

**Authors:** Yanina de Lafuente, Eride Quarta, María S. Magi, Ana L. Apas, Joaquín Pagani, María C. Palena, Paulina L. Páez, Fabio Sonvico, Alvaro F. Jimenez-Kairuz

**Affiliations:** 1 Departamento de Ciencias Farmacéuticas, Facultad de Ciencias Químicas, Universidad Nacional de Córdoba (UNC), Córdoba X5000HUA, Argentina; yanina.de.lafuente@unc.edu.ar (Y.d.L.); maria.sol.magi@unc.edu.ar (M.S.M.); aapas@unc.edu.ar (A.L.A.); joaquin.pagani@mi.unc.edu.ar (J.P.); celeste.palena@unc.edu.ar (M.C.P.); plpaez@unc.edu.ar (P.L.P.); 2 Unidad de Investigación y Desarrollo en Tecnología Farmacéutica (UNITEFA), Consejo Nacional de Investigaciones Científicas y Técnicas (CONICET-UNC), Haya de la Torre y Medina Allende, Ciudad Universitaria, Córdoba X5000HUA, Argentina; 3 Department of Food and Drug Science, University of Parma, Parco Area Delle Scienze 27/A, 43124 Parma, Italy; eride.quarta@unipr.it (E.Q.); fabio.sonvico@unipr.it (F.S.)

**Keywords:** pulmonary infection, polyelectrolyte–drug complex, inhaled tobramycin, delivery system, *Pseudomonas aeruginosa*, *Staphylococcus aureus*

## Abstract

**Background/Objectives:** Pulmonary delivered tobramycin (TOB) is a standard treatment for Pseudomonas aeruginosa lung infections, that, along with Staphylococcus aureus, is one of the most common bacteria causing recurring infections in CF patients. However, the only available formulation on the market containing tobramycin, TOBI^®^, is sold at a price that makes the access to the treatment difficult. Therefore, this work focuses on the development and characterization of an ionic complex between a polyelectrolyte, hyaluronic acid (HA) and its salt, sodium hyaluronate (NaHA), and TOB to be formulated as an inhalable dry powder. **Methods:** The solid state complex obtained by spray drying technique was physicochemically characterized by infrared spectroscopy, thermal analysis and X-ray diffraction, confirming an ionic interaction for both complexes. **Results:** The powder density, geometric size, and morphology along with the aerodynamic performance showed suitable properties for the powder formulations to reach the deep lung. Moisture uptake was found to be low, with the complex HA-TOB remaining physicochemically unchanged, while the NaHA-TOB required significant protection against humidity. The biopharmaceutical in vitro experiments showed a rapid dissolution which can have a positively impact in reducing side effects, while the drug release study demonstrated a reversible polyelectrolyte–drug interaction. Microbiological experiments against *P. aeruginosa* and *S. aureus* showed improved bacterial growth inhibition and bactericidal efficacy, as well as better inhibition and eradication of biofilms when compared with to TOB. **Conclusions:** A simple polyelectrolyte-drug complex technique represents a promising strategy for the development of antimicrobial dry powder formulations for pulmonary delivery in the treatment of cystic fibrosis (CF) lung infections.

## 1. Introduction

Cystic fibrosis (CF) is an autosomal recessive disease that affects 1 in every 2000 to 4000 live births [1,2]. This genetic disorder renders CF patients susceptible to chronic lung infections, which are the most common form of clinical presentation and the leading cause of morbidity and mortality. In the lungs, the presence of viscous secretions results in the development of progressive obstruction, chronic inflammation, and long-term damage to the airways. This, in turn, leads to a decreased ability to clear secretions, resulting in increased infection rates [3].

The most common bacterial species responsible for recurrent infections are *Staphylococcus aureus* and *Pseudomonas aeruginosa*, which are treated systemically with antimicrobials administered intravenously or orally [4]. However, the inhalation route is preferred for local treatment. Inhaled antibiotics represent a pharmacotherapeutic alternative for the treatment of chronic respiratory tract infections, as they allow high drug concentrations to be achieved in the lungs, while minimizing the risk of systemic side effects and increasing patient compliance [5]. Tobramycin (TOB) inhalation is used in CF patients to treat lung infections caused by *P. aeruginosa* [6]. Currently, there is one product approved containing TOB for the treatment of infections caused by *P. aeruginosa*, i.e., TOBI PODHALER^®^ inhalation powder (Viatris, Canonsburg, PA, USA). However, the cost of these dry powder inhalers (DPI) may limit patient accessibility. Despite this, DPIs offer several advantages over aerosol and nebulizer formulations: they are easy to use, portable, and eliminate the need for propellants. In addition, dosing is not influenced by patient coordination. However, they require the formulation of solid particles that are able to access the areas of the lung where the drug (D) will exert its action. In this context, particle size, morphology, and density are of significant importance, as these parameters will determine whether the particles reach the site of action or are swallowed or exhaled [5].

Spray drying is a technique that is widely used in the pharmaceutical industry. It is considered attractive due to its rapid, continuous, reproducible nature and the fact that it can be scaled up without major modifications. Currently, spray drying is one of the preferred methods for the production of inhalable powders due to its ability to control of the final particle’s properties and its cost-effectiveness. It is a fast, one-step method with an optimal yield and high reproducibility [7,8]. The development of carrier-free DPI has become an active area of research in the last decades. The main challenge is to tailor the physicochemical characteristics of microparticles, including crystallinity, surface free energy, size, density, and shape [9]. In this context, a particulate inhaled formulation based on a co-processed material prepared by the spray drying an acid–base complex between vancomycin and hyaluronic acid (HA) was recently obtained, with aerodynamic performance suitable for pulmonary administration [10]. The acid–base interaction between the carboxylic groups of a polyelectrolyte (PE) and a basic group of a drug results in a high degree of counterionic condensation. Polyelectrolyte–drug (PE-D) complexes exhibit several unique and favorable properties that can be exploited in the design of smart or stimuli-responsive drug delivery systems [11,12,13,14]. Furthermore, as PEs are mucoadhesive, they bind to physiological mucous membranes, increasing the residence time of the drug, allowing its permeation across biological barriers or prolonging its local action, greatly improving the performance of the treatment [15,16,17].

Consequently, pharmaceutical technology platforms based on PE-D complexes represent a good strategy for the development of inhaled powder formulations through a straightforward and scalable process. The HA, and its salt, the sodium hyaluronate (NaHA) chosen as the PE carrier are pharmaceutical excipients recognized as safe (GRAS classification, FDA USA) and widely used for therapeutic, restorative, cosmetic, or nutritional purposes [18]. They are hygroscopic polymers capable of retaining large amounts of water in the extracellular matrix, forming gels that promote tissue homeostasis and integrity and provide mucus-fluidizing properties [19].

Recent reports indicate that the properties of HA are determined by its molecular weight. High-molecular-weight HA has been shown to have an anti-inflammatory effect [20]. This process is due to the fact that HA is able to hydrate the airways and repair the extracellular matrix, reducing inflammation and attenuating bronchial hyperresponsiveness [21,22]. In this context, medicinal products containing HA for nebulization are available (NEOSTEX Respire^®^, Neochemical, Avila Spain; HYANEB^®^, Chiesi Pharmaceuticals, Parma, Italy), which are indicated for mucus thinning in patients with lung diseases such as CF.

The mucoadhesiveness and anti-inflammatory effect of the co-processed material, in addition to the potential for the generation of formulations free of additional excipients with optimal aerosolization properties, renders it an appealing strategy for the development of inhaled pharmaceuticals with enhanced characteristics in comparison to available pharmacological therapies.

In this context, the aim of this study was to develop and characterize a particulate inhaled formulation combining TOB and HA/NaHA with optimized aerosolization behavior by spray drying and demonstrating its antibacterial properties against *P. aeruginosa* and *S. aureus.*

Since the acidic form of HA is not commercially available and must be obtained from the acid–base neutralization of sodium salt dispersions, it was also an objective to compare the products obtained from the drug co-processing with HA, i.e., the acid form or its sodium salt, NaHA.

## 2. Results and Discussion

### 2.1. Complexes Preparation

The use of HA or its salt, the NaHA, as PE is not only useful for the capability of forming complexes due to the ionizable groups but also because it plays an important role in the lungs, since it has been found to be an anti-inflammatory polymer at high molecular weight [23]. The difference between HA and NaHA may influence the selection of one over the other. While the HA has a higher number of ionizable groups to interact with the TOB, therefore, having a major neutralization percentage, the NaHA does not require acid–base neutralization to be prepared, which simplifies the manufacturing process.

Different neutralization ratios were tested based on PE-D technology, yielding similar results across the various neutralization percentages of PE with TOB. Since the different neutralization percentages showed good results, the highest percentage was chosen for its ability to deliver the greatest amount of TOB, which would reduce the required drug dosage.

The respective raw materials of the complex HA-TOB were easily dispersed in water, and the pH was corrected without visible changes in its aspect. The dispersion of NaHA required a slightly greater amount of time than HA, since it produced a solution with higher viscosity. However, the mixing of the NaHA and TOB dispersions produced a homogeneous stable suspension of the complex, which lasted at least until the obtention of the powder by spray drying. The resulting powders exhibited a white color, and the yields for HA-TOB and NaHA-TOB were 57.1 ± 0.2 and 45.0 ± 6.0%, respectively. The lower yield for the complex NaHA-TOB could be related to the higher adhesion observed in the spray dryer’s walls.

### 2.2. Physicochemical Characterization of HA-TOB and NaHA-TOB in the Solid State

The solid-state characterization of HA-TOB and NaHA-TOB microparticles compared with their precursors was carried out using different techniques, such as FT-IR analysis, PXRD, TGA, and DSC.

**Figure 1:**
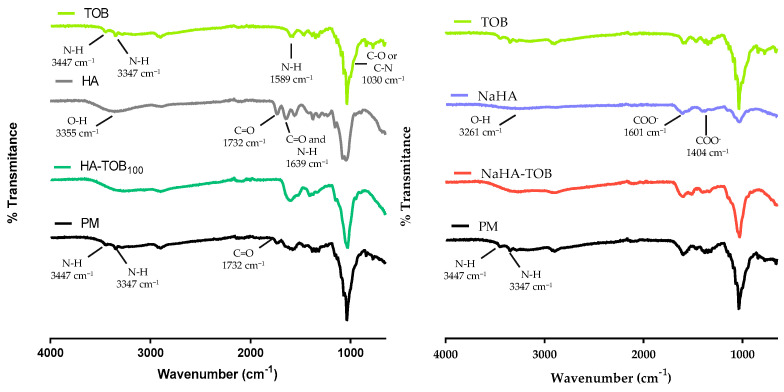
Overlapped Fourier transform infrared spectroscopy spectra of HA-TOB and NaHA-TOB complexes; raw materials (tobramycin (TOB), hyaluronic acid (HA), and sodium hyaluronate (NaHA)) and physical mixtures (PM) of both complexes.

Figure 1 shows the FT-IR spectra of both complexes (HA-TOB and NaHA-TOB), their precursors, and their PMs in order to study the ionic interactions that would occur due to the acid–base interaction between the PE and the oppositely charged drug. As can be seen in Figure 1, in the HA spectrum, an extensive band at 3355 cm^−1^ is observed and could be attributed to the O-H tensile vibration of the COOH group and other two bands at 1732 cm^−1^ and 1639 cm^−1^ corresponding to the C=O carbonyl stretching vibration of the COOH group are also present [24,25]. On the other hand, in the NaHA spectra, it is possible to see the same band around 3261 cm^−1^ corresponding to the O-H tensile vibration of the COOH group, but the other two bands present in HA have disappeared. Instead, other bands at 1601 cm^−1^ and 1404 cm^−1^ appeared and could be related to the symmetric and asymmetric vibration of the ionized carboxyl group. It is important to note that in NaHA, the carboxyl group is deprotonated (COO^−^) to form the salt with Na^+^, which is consistent with the bands observed in its spectrum [24].

Regarding TOB spectrum, two bands at 3447 cm^−1^ and 3347 cm^−1^ can be observed, which correspond to the stretching vibration of the N-H group and are characteristic of primary amines. These bands are followed by another one at 1589 cm^−1^ that may be attributed to the N-H vibration deformation. Additionally, a band present at 1031 cm^−1^ could correspond to the C-O or C-N stress vibration of the amine group [24,26,27].

In the spectra of HA-TOB, the band at 1732 cm^−1^ corresponding to the C=O vibration of COOH present in HA, as well as the bands at 3447 cm^−1^ and 3347 cm^−1^ attributed to the stretching vibration of the N-H group present in TOB, disappeared. Conversely, these bands are presented in the PM spectrum. These changes in the complex spectrum suggested a clear indication of acid–base interaction between HA and TOB to form HA-TOB. These findings are in agreement with other complexes with HA reported in the literature [10,24].

In the case of NaHA-TOB, the band at 1732 cm^−1^ cannot be used as an indicator of the evidence of complex formation, because it was absent in the NaHA spectrum, but the disappearance of the bands at 3447 cm^−1^ and 3347 cm^−1^ of the primary amine group in its neutral form, which were present in its PM spectrum, could suggest a potential ionic interaction between NaHA and TOB.

**Figure 2:**
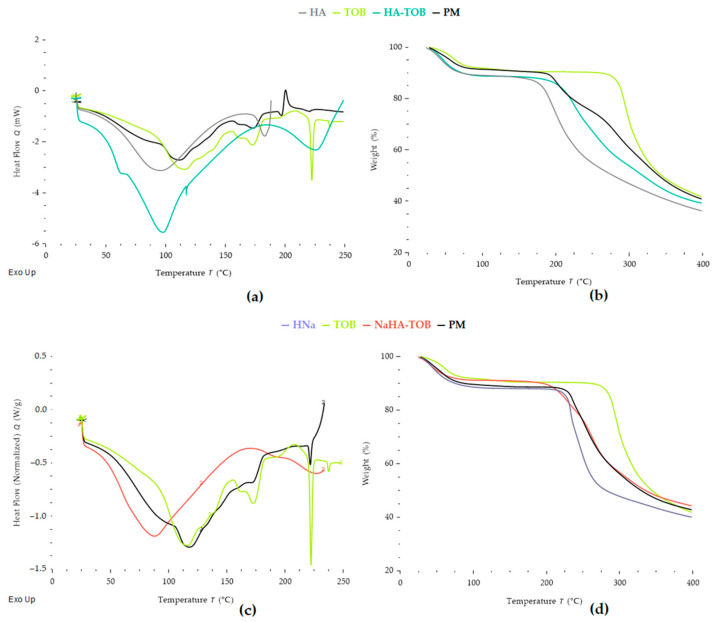
Thermal analysis by differential scanning calorimetry (DSC) (**a**) and thermogravimetric analysis (TGA) (**b**) of the complex HA-TOB, its raw materials, and the physical mixture (PM). DSC (**c**) and TGA (**d**) of the complex NaHA-TOB, its raw materials, and the PM.

DSC and TGA thermograms, depicted in Figure 2, showed the thermal characteristics of HA-TOB, NaHA-TOB, their precursors, and their PMs in order to determine not only the thermal stability of each independent material but also the changes produced by interactions due the formation of the complex [28,29,30].

The DSC curve of HA exhibits a broad endothermic peak, from room temperature to 92 °C, corresponding to the loss of water adsorbed to PE with a weight loss of 11%, in the TGA [31,32]. Additionally, a second endothermic peak, followed by the onset of an exothermic peak at approximately 182 °C matched by mass loss in TGA, was attributed to the decomposition of HA [10]. This behavior has been reported by other authors, who described a multi-stage decomposition process for HA, where the first degradation step involves dehydration from the compound structure, followed by a decomposition stage near 200 °C [32]. In turn, other findings report an average decomposition temperature for HA around 240 °C [31,33,34]. The glass transition (Tg) of pure HA could not be observed under the conditions applied. The literature on this subject is disparate among authors. While some claim that HA has a Tg that is around room temperature (20–25) °C [35], others report Tg values below 0 °C, between (−48 and −80) °C, which are inversely proportional to the adsorbed water content [36].

Regarding the thermal behavior of TOB, two slightly overlapping peaks appear when the weight loss is analyzed by TGA, one at 74.8 °C with a weight loss of 8.3% and the second at 102.9 °C with a loss of 1.90%. In addition, a broad endothermic peak starting from room temperature with a peak at 113 °C is observed by DSC. These events suggest that TOB was in the trihydrate form at the start of the assay and its dehydration occurs in two stages, forming a metastable anhydrous form [37]. After dehydration, TOB showed three peaks in its DSC curve without any weight loss in the TGA, which, according to the literature, correspond to the melting of the metastable form and recrystallization, culminating in a melting peak. The first endothermic peak is present at 171.4 °C and corresponds to the melting point of the metastable form of TOB. A second exothermic peak, poorly defined, concerning the recrystallization of the anhydrous metastable form, appears at 211 °C, and, finally, a narrow endothermic peak is observed at 221.4 °C due to the melting of the stable TOB in its crystalline form [37,38,39]. Finally, at a temperature of 284 °C, a sustained weight loss is observed and can be attributed to the decomposition of TOB.

When comparing HA-TOB with the corresponding PM, major differences are observed. It can be firstly highlighted that the complex HA-TOB, as well as TOB and its PM, presented two-stage dehydration. However, there was a shift in their dehydration temperatures: 74.3 °C and 107.7 °C for the PM, with events occurring at temperatures similar to TOB alone, compared to 59.3 °C and 90.80 °C for HA-TOB. This shift may be related to the interaction between TOB and HA. Additionally, the similarity of DSC thermograms of PM and pure TOB can be clearly observed, with the exothermic peak associated with TOB re-crystallization being more defined, and the melting peak less evident, which could be related to the presence of HA, that acts as an impurity. The decomposition of PM occurred in two phases, the first at 202.3 °C and the second at 292.3 °C, which could correlate to the decomposition of HA and TOB, respectively, and it is indicative that the PM curves present the thermal characteristics of its precursors. Conversely, in the HA-TOB complex, these characteristic peaks related to the melting of the TOB metastable form and the crystallization of the anhydrous form were not present in the thermogram or were so poorly defined that they overlapped with the dehydration phenomenon. This thermal behavior suggests that the majority of the TOB was interacting with the HA and was not able to crystallize. Finally, a decomposition is observed for HA-TOB at a temperature of 233 °C, which, unlike its respective PM, occurs in a single phase. The same behavior was observed when comparing NaHA-TOB and its PM, where the last one showed the sum of NaHA and TOB peaks. For NaHA-TOB, it was not possible to distinguish a two-stage dehydration, but only one, being an endothermic process that has its minimum at 84 °C, while the PM presented a two-stage dehydration at 84.7 and 116.4 °C. After dehydration, the complex NaHA-TOB only exhibited a decomposition endotherm at 229.6 °C. Additionally, it can be highlighted that the stability of TOB was not compromised by the interactions with HA or NaHA since the decomposition temperatures are above the working temperatures, as well as the temperature used to obtain the solid by the spray-drying process, giving it the necessary thermal stability for its use and handling.

**Figure 3:**
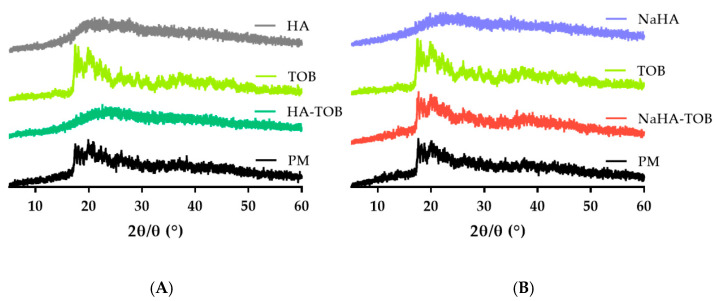
Powder X-ray diffraction patterns of the HA-TOB, its precursors, and its PM (**A**) and of the NaHA-TOB, its raw materials, and its PM (**B**).

Complementary PXRD patterns of TOB, HA, NaHA, both complexes, and its PM were carried out. As can be seen in Figure 3, HA and NaHA presented an amorphous solid diffraction pattern, evidenced by the absence of peaks, and this characteristic has been previously reported [10]. On the other hand, TOB showed a diffractogram without a sharp peak, but with some defined peaks at 2ϴ values between 17° and 26°, in accordance with other authors [37,38,40]. In both PMs, the same series of defined peaks were observed, but with a lower intensity, possibly due to the presence of an amorphous solid, HA or NaHA, which has an impact in the obtained pattern. Interestingly, in the diffractogram of HA-TOB, these peaks disappear and an amorphization of the solid is observed, i.e., the TOB loses its characteristic semi-crystalline diffractogram due to an interaction with HA. This rearrangement at the molecular level is indicative of a successful PE-D interaction and has been previously observed [41,42,43]. However, when the NaHA-TOB pattern was analyzed, it retained the characteristic peaks of TOB as well as its PM, perhaps due to a weaker or incomplete interaction between NaHA and TOB due to the presence of the sodium counterion. This displacement in the ionic complex, as evidenced by the crystalline peaks in PXRD, might not be sufficient to observe the melting peak by DSC (Figure 2)

The available inhaled TOB, TOBI PODHALER^®^, presents an amorphous state [44] like HA-TOB, and this structure is preferred over a crystalline one for pulmonary drug delivery, because it offers several advantages [45], while crystalline solids could lead to local adverse effects due to the permanence of the undissolved solid, such as throat irritation and an inflammatory response [46,47,48,49].

### 2.3. Powder Characterization

Bulk (δ_B_) and tapped (δ_T_) densities are parameters that could affect the aerosolization properties of the dry powder and the delivery of the drug from the inhaler device to the lungs [50]. The density values were measured and based on these, CI and HR were determined as an indication of flowability properties; the results are present in Table 1. According to the values obtained, both complexes showed δ_T_ below 0.4 g/cm^3^, indicating that it is appropriate for powder aerosolization and deposition to the deep lung [51,52,53]. A low density can lead to a low flowability [53]. In fact, our results of CI and HR indicated that the flowability is very poor according to the USP [54]. Good flowability properties could help to optimize the emptying of the dose from the inhaler and facilitate the filling of the capsule, whereas the low density could allow better transportation through the airstream produced during inhalation [53,55]. Therefore, a balance is necessary to achieve good deep lung delivery as well as good powder emission. It is important to note that δ_T_ is not the only factor responsible for the flowability but also other parameters could affect it, such as the size, moisture content, porosity, and morphology of the particles [53,56].

**Figure 4:**
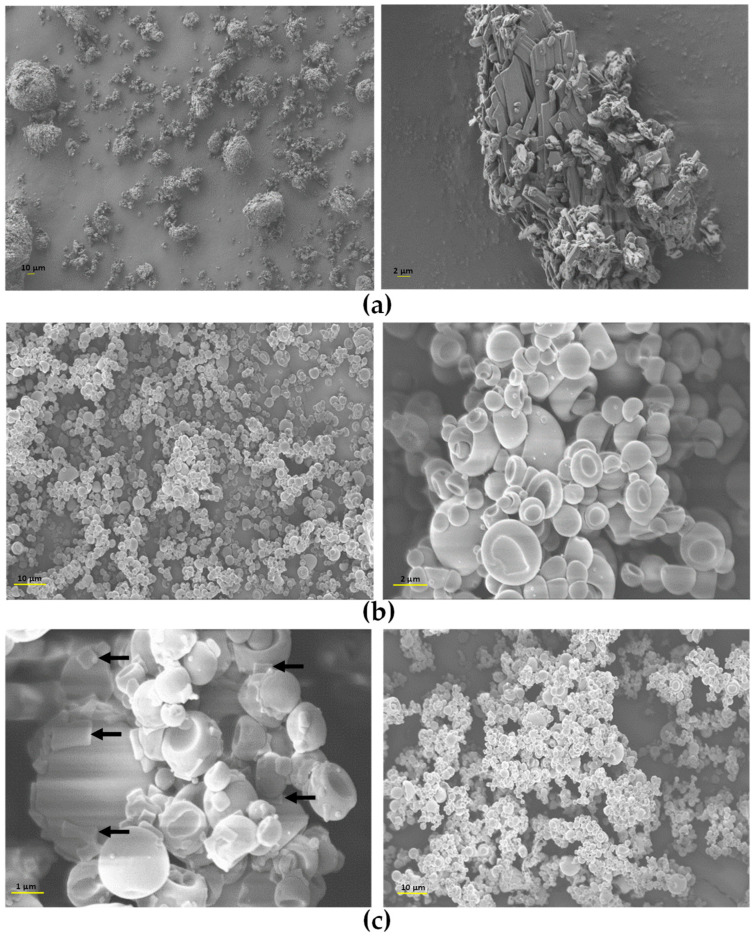
Scanning electron microscopy (SEM) micrographs of TOB (**a**), HA-TOB (**b**), and NaHA-TOB (**c**) obtained by spray drying with magnifications of 500×, 1000×, 5000×, and 10,000×. The arrows indicate the deposition on the particles of crystalline non-complexed TOB.

Regarding the morphological properties of powders, images were taken by SEM and are shown in Figure 4. TOB, the pure drug, presents bladed crystals with a wide size distribution agglomerated in large clusters, which would deposit in the upper airways and could lead to throat irritation if inhaled without changes. In addition, the agglomerates observed in the images, due to their large particle size, may hinder pulmonary delivery altogether by preventing the particles from reaching the deep lung. Instead, they are likely to deposit in the upper airways by inertial impaction, reducing the therapy effectiveness [57]. Conversely, HA-TOB and NaHA-TOB particles showed a spheroidal and hollow or collapsed morphology with a smooth surface, in some cases with particles inside other particles, as previously described by other authors [58,59]. This morphology observed in both systems may be the reason for the low density described above and can be attributed to the spray-drying technique [57], which also allowed the obtaining of a powder with narrower particle size compared to TOB alone [60]. The only difference between the two complexes, when observed at the higher magnification, was the presence in the SEM micrographs of NaHA-TOB of few potentially crystalline structures, which could be attributed to non-complexed TOB. This result is consistent with the PXRD pattern previously described, where the complex with NaHA showed the characteristic peaks of TOB. On the contrary, the absence of any particle with a different morphology in the HA-TOB image could imply a complete interaction between HA and TOB.

As can be seen in Figure 4, both complexes presented a small size, which increases the contact area of the particles. The smooth surface, devoid of any roughness that separates them from the rest of the particles [61,62], produced an increase in particle–particle interaction given predominantly through Van der Waals type and also electrostatic forces [56,63,64,65]. This causes powder cohesiveness and leads to low flowability and agglomeration, as observed macro- and microscopically [66]. The observed adhesion and agglomeration can also be attributed by the collapsed surface that induces a mechanical blockage by the larger particles on the smaller ones [65].

Particle size is one of the most relevant aspects to be considered in the development of formulations administered by the inhaled route. Particles larger than 5 µm will not reach the deep lung to exert their action, while particles smaller than 0.5 µm might be exhaled during expiration [52,57,67]. Table 2 presented the Dv10, Dv50, Dv90, and SPAN values obtained for HA-TOB and NaHA-TOB. As can be seen, both complexes exhibited a Dv50 of 2.9 and 4.14 µm for HA-TOB and NaHA-TOB, respectively, which is an indication that the average particle size of the powders is appropriate for deep lung deposition. Additionally, it is noticeable that the complex with HA presents a smaller size than the complex with NaHA.

Regarding the distribution width, the SPAN values must be < 2.0 to be considered a narrow, uniform, and monodisperse distribution. HA-TOB complies with the expected value of SPAN, whereas NaHA-TOB is located on the borderline, and as a result, an improved control of pulmonary deposition and aerosolization performance which can reduce dose variability is expected.

The presence of water in a solid formulation can lead to increased chemical and/or physical instability, especially when the formulation is exposed to high RH for extended periods of time, as is common during manufacturing, storage, and use of the product [56]. It also could produce changes in its morphology, an increase in bulk density, a decrease in flowability, and the formation of irreversible aggregates. These changes can negatively impact the emission performance of the inhalable powder from the capsule as its subsequent aerosolization, which is crucial in the development of inhaled formulation [52,63,68,69].

**Figure 5:**
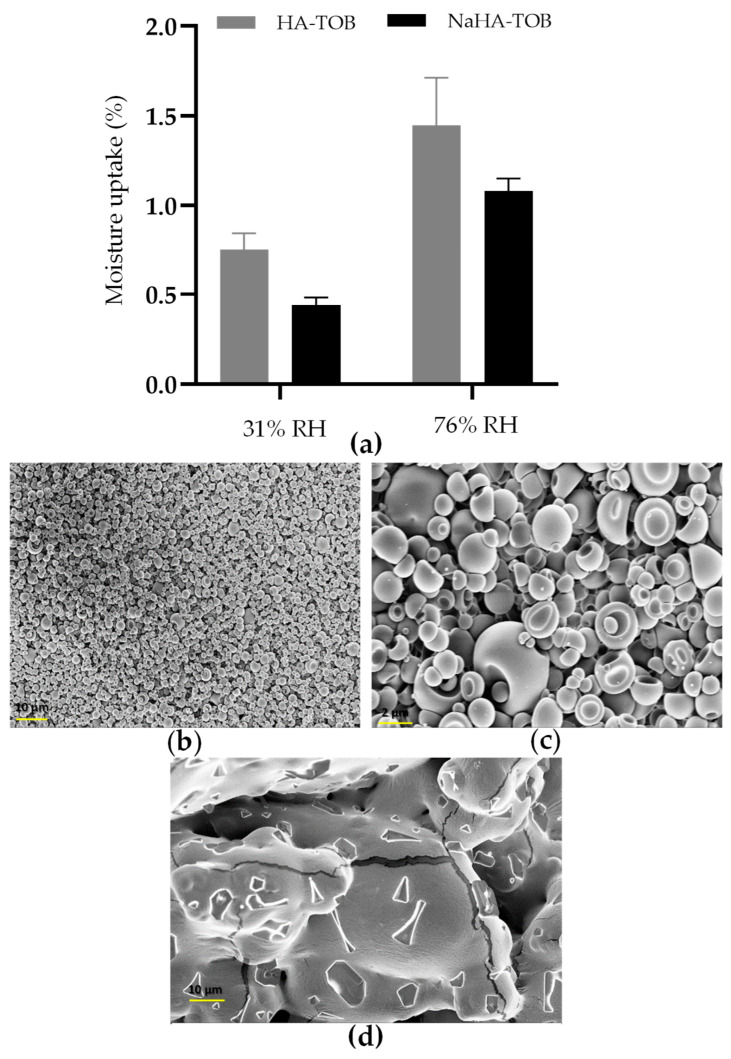
Moisture uptake of the complexes stored at 25 °C and a relative humidity (RH) of 31 and 76 (**a**). SEM micrographs of HA-TOB at a magnification of 1000× (**b**) and 5000× (**c**), and NaHA-TOB at a magnification of 1000× (**d**) after storage at 25 °C and 76% HR in a sealed container.

In this sense, a study of the moisture uptake of the materials was performed at room temperature under two different RHs, 31 and 76%. Prior to this, powders were dried out to ensure that any water present was due to the adsorption or absorption of ambient moisture rather than residual water. Figure 5 showed the percentage moisture content of HA-TOB and NaHA-TOB after reaching equilibrium. It can be observed that both complexes exhibited a low moisture content, with values lower than 1% at 31% RH and increasing up to 1.5% at 76% RH. These are promising results since the low moisture content values were achieved despite the presence of HA or NaHA and without the need to add an excipient such as mannitol, which has a crystalline form with low hygroscopicity, and is often added to formulations to keep water content low [70]. Comparing both complexes, it is possible that NaHA-TOB resulted in lower moisture uptake than HA-TOB, possibly due to its semi-crystalline form, as demonstrated by PXRD. It is known that, generally, an amorphous material has a greater hygroscopic capacity than a crystalline material [71].

After exposure to an RH of 76%, no macroscopic alterations due to the increase in the moisture content were observed in both powders, maintaining their whitish appearance. However, to study the microscopic appearances, SEM micrographs were taken and are displayed in Figure 5. As can be seen, while HA-TOB presented the collapsed spheroidal shape with a smooth surface that was previously described, NaHA-TOB showed evident changes in its morphology, becoming a single united mass that increased considerably in size. The moisture uptake could accelerate or trigger a degradation reaction of TOB in the NaHA-TOB complex, leading to the formation of irreversible agglomerates which ultimately affected its morphology. Oxidation has been described as the main degradation mechanism of TOB in solutions and occurs mainly at neutral pH values as the pH of the formulations where the NaHA-TOB may only be affected due to the presence of non-interacting TOB, as previously detailed [72,73]. Another hypothesis may be that moisture favors the massive crystallization of displaced TOB from NaHA. Although the effect of humidity over time under real storage conditions was not studied, it can be anticipated that the NaHA-TOB complex would be affected due to the presence of non-interacting TOB, as previously mentioned. This would require enhanced protection against both humidity and temperature.

### 2.4. In Vitro Biopharmaceutical Performance

The aerodynamics performance of the complexes was determined based on the TOB content deposited at each stage of the NGI, through quantification by HPLC. Table 3 shows the values of the aerodynamic parameters obtained for both inhalable powders, HA-TOB and NaHA-TOB. A high EF can be observed for both complexes, beyond the 75% USP limit [74]. As reported in Table 3, NaHA-TOB and HA-TOB presented values of FPF of about 60% and 52%, respectively, without significance difference (*p* > 0.05). Hence, more than 50% of the particle’s population of both powders will deposit in the bronchial region, as they have an aerodynamic diameter <5 µm, while 25% of the NaHA-TOB and 20% of the HA-TOB will reach the alveolar region due to their size < 3 µm. Finally, the MMAD represents the size that 50% of the population exhibits. According to the obtained data, the MMAD of both complexes was 2.5 and 2.6 µm for NaHA-TOB and HA-TOB, respectively.

The EF values obtained suggest that the aggregates observed macro- and microscopically mentioned above do not present strong or irreversible intermolecular forces, and they were de-agglomerated during aerosolization through the inhaler. Furthermore, the low flowability previously described for both powders was not a limiting factor for accurate dosing.

**Figure 6:**
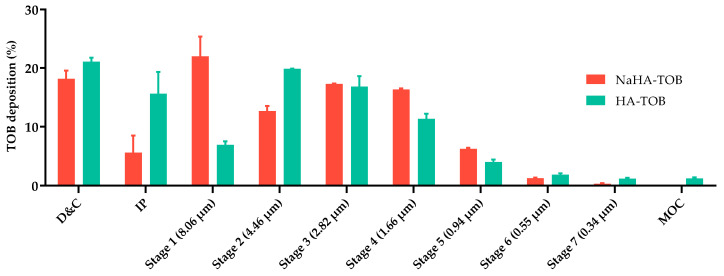
TOB deposited and recovered from the inhaler device and capsule, and the different stages of the NGI of the HA-TOB and NaHA-TOB.

Whereas the objective of our systems is to be useful as a local treatment, it is known that pulmonary inflammation and excessive mucus production in CF patients leads to a decrease in airway caliber, making a small aerodynamic size even more relevant to achieve drug delivery to the entire bronchial tree. Otherwise, it is even more likely that particles’ powder will be deposited in the upper airways due to sites of obstruction in the periphery of the airways [75,76,77,78,79]. The MMAD values obtained in this work for both TOB powders, consistent with previous studies reporting MMADs between 2 and 3 µm, may be more beneficial for CF patients [79]. It can be observed in Figure 6 that most of the powder was deposited between stage 1 and stage 5 of the NGI for NaHA-TOB and stage 2 and stage 5 for HA-TOB.

The dissolution process of the powder deposited in the lungs depends on the deposition location as well as the physicochemical properties of the powder’s particles. Despite the lack of standardized methods on pharmacopeias and guidelines for inhalable powders, the dissolution test is an important tool in pulmonary drug delivery for development formulation in the inclusion or exclusion of excipients [80]. Due to the noticeable difference between oral and pulmonary drug delivery, it is relevant to have a dissolution method that reflects these contrasts. The Franz cells can be used as an alternative methodology, as their volume of approximately 16 mL is closer to the 10–30 mL of the lung lining fluid than the 900 mL typically used in oral dissolution methodologies. Additionally, this method provides an air–liquid interface which replicates the physiological conditions of the lungs. The conventional procedure of the use of the Franz cells can present a greater degree of complexity, particularly with regard to the differentiation of the dissolution from the diffusion rate. Hence, instead of using a semipermeable membrane between the donor and the receptor compartment of the cells, a cellulose filter was employed [81,82,83].

**Figure 7:**
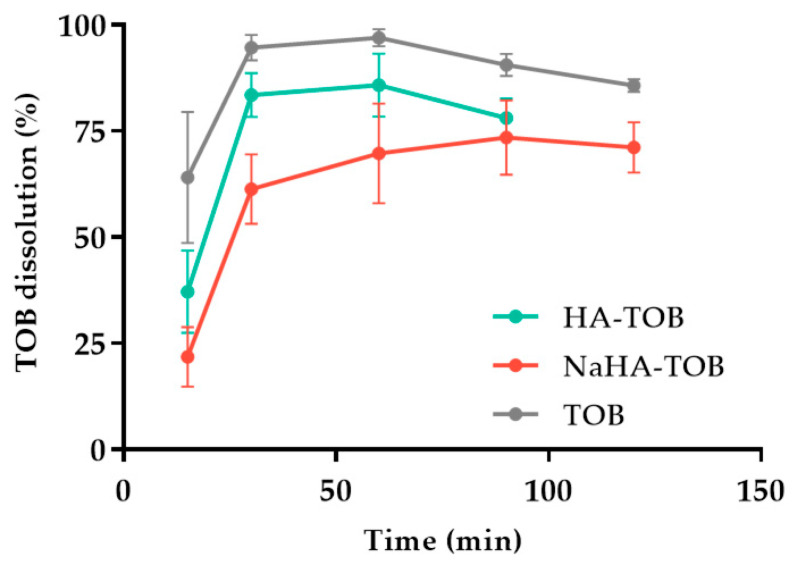
Dissolution profiles of the complexes HA-TOB and NaHA-TOB, and the drug (TOB).

The dissolution profile of TOB, HA-TOB, and NaHA-TOB is depicted in Figure 7, showing differences between all of them (*f*_2_ < 41.0). As it was expected, the TOB, without a PE interaction, presented the fastest dissolution profile, followed by HA-TOB and NaHA-TOB with 94.6%, 83.4%, and 61.3% dissolution percentages in the first 30 min of the experiment, respectively. The PE presence in contact with the dissolution media might be producing the gelation and swelling, causing the reduction in dissolution speed presented below. Furthermore, the previously shown semi-crystalline state of the NaHA-TOB can be the reason why the dissolution profile of the respective complex occurred to be slower than the amorphous complex HA-TOB. Nevertheless, the fast dissolution rate obtained for both complexes is an important aspect that can possibly have an impact in the reduction in side effects, such as irritation of the throat, related to persistent residual particles in the surface of the lung epithelium, and just as well, the reduction in macrophage phagocytosis and/or mucociliary clearance, avoiding a fast drug removal from the lungs [47,84,85].

### 2.5. Microbiological Activity Tests

As mentioned above, *P. aeruginosa* and *S. aureus* are the most common bacteria infecting the lungs in patients diagnosed with CF, and these bacteria have the ability to form biofilm, which leads to persistent and chronic infections deteriorating the lung function [86,87].

The assessment of the antimicrobial efficacy of the two complexes, HA-TOB and NaHA-TOB, in comparison with the drug, TOB, against the *P. aeruginosa* and *S. aureus* strains, was realized by determining the MIC and the MBC, presented in Table 4. The data obtained showed antimicrobial activity for TOB and the complexes against both strains. It is no less important to remark that the complexation of the TOB with HA or NaHA did not affect the recognized activity of TOB; quite the opposite, as both complexes required less of the drug to achieve similar results than the TOB used as a reference, presenting lower MIC and MBC values. As seen in previous reports, the improved and sustained antibacterial activity can be attributed to the slow and controlled release of TOB from the complexes in the bacterial environment, due to the high counterionic condensation. This property, together with the mucoadhesive nature described for HA, allows us to predict good in vivo performance [88].

These findings can be beneficial, as they allow a lower antimicrobial dose administration, reducing the risk of adverse effects and the development of bacterial resistance.

The ability of these bacteria to protect themselves by forming a biofilm leads to the importance of investigating the capability of the complexes to inhibit biofilm formation or to eradicate the mature biofilm. Inhibition and eradication studies of the biofilm produced by *P. aeruginosa* and *S. aureus* were carried out. Regarding the biofilm inhibitory activity of both complexes against the *P. aeruginosa* strain, it was observed that they exhibited a higher inhibitory effect in comparison to the drug, starting from high concentrations (250-fold times the MBC value) down to diluted concentrations (1–0.5-fold times the MBC value). In contrast, TOB demonstrated an inhibition of approximately 50% at concentrations 15.6 times its MBC value. Conversely, for the eradication of mature *P. aeruginosa* biofilm, the HA-TOB complex exhibited superior efficacy in comparison to NaHA-TOB and TOB, achieving eradication above 50% over a wide concentration range, even at 0.5 times its MBC value. Conversely, NaHA-TOB exhibited minimal efficacy, failing to attain more than 50% eradication within the examined concentration range.

In relation to the inhibition of the biofilm produced by *S. aureus*, a similar pattern was observed for both complexes and TOB, showing a higher inhibition for the complex HA-TOB at lower concentrations near its MBC value. It was remarkable that the complex HA-TOB reduces the biofilm formation by 50% in a range of concentrations from 500 to 0.25-fold times its MBC. Furthermore, the eradication of the mature biofilm produced by *S. aureus* showed similar results for both complexes and TOB.

It is worth highlighting the results obtained by the HA-TOB complex in the inhibition and eradication of the biofilm produced by the bacteria that most frequently infect the airways of CF patients. This allows not only the prevention of biofilm formation but also the disruption of the already formed biofilm which is critical in chronic infections.

**Figure 8:**
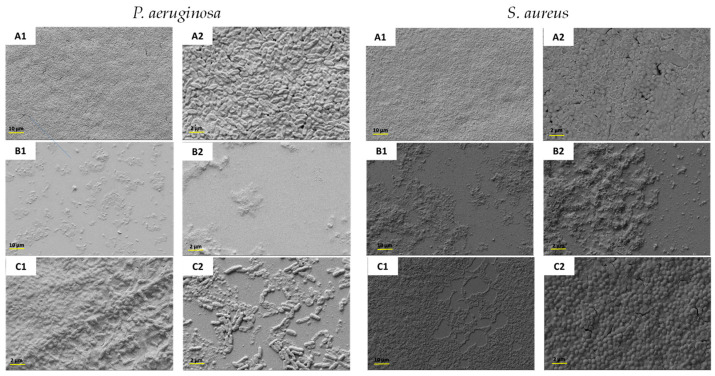
SEM micrographs at a magnification of 1000× (1) and 5000× (2) of the biofilm produced by *P. aeruginosa* and *S. aureus* not treated (**A1**,**A2**) and treated with HA-TOB to evaluate the inhibition (**B1**,**B2**) and eradication (**C1**,**C2**) of the respective strains.

Owing to the observed biofilm inhibition and eradication behavior, the most promising complex, HA-TOB, was subjected to SEM analysis to elucidate the preceding results. Figure 8 shows representative images of the biofilm growth (control) and the inhibition and eradication caused by the complex. The micrograph corresponding to the control of both strains showed the typical bacillus and coccus morphology of *P. aeruginosa* and *S. aureus*, respectively, accompanied by discernible biofilm formation. In the study of the *P. aeruginosa* biofilm, as was expected, the complex produced a visible reduction in biofilm formation as well as a disruption of the mature biofilm. For both cases, the morphology of the bacillus changed to a flat appearance with significant damage to the bacterial walls. A similar, but less marked, effect was observed in the inhibition of *S. aureus*, where a decrease in the biofilm formation was seen, as was a less noticeable biofilm eradication.

The data suggest that the interaction between the TOB and the respective PEs (HA and NaHA) improves the biofilm inhibition and eradication against both bacterial strains, especially the complex HA-TOB, compared to TOB alone, which has been described as having limited activity against the biofilm produced by *P. aeruginosa* [89,90]. This can be attributed to the HA antiadhesion activity as well as an antibiofilm property, which facilitate penetration into the bacterial biofilm or disrupt its integrity, thereby enhancing antimicrobial activity, as reported by other researchers [91,92,93].

## 3. Materials and Methods

### 3.1. Materials

Tobramycin (Unifarma, V-120601, Córdoba, Argentina), high-molecular-weight sodium hyaluronate (NaHA) (Pura Química^®^, average MW 1.0–2.0 MDa, batch n° 313318, Córdoba, Argentina), cation exchange resin (Amberlite^®^ IR120 Fluka, Sigma-Aldrich, St. Louis, MO, USA), NaCl, KCl, NaHPO_4,_ and KH_2_PO_4_ (Parafarm^®^, Buenos Aires, Argentina), NaOH and HCl 1N (analytical reagents, Anedra, Córdoba, Argentina), acetonitrile (VWR International, Fontenay-sous-Bois, France), dimethyl sulfoxide (DMSO, Anedra, Buenos Aires, Argentina), Tris (hydroxymethyl) aminomethane (Biopack^®^, Buenos Aires, Argentina), and Müeller Hinton broth and agar (Britania^®^, Buenos Aires, Argentina) were used as provided by the suppliers. Ultrapure water (water purification NW-system, Heal Force Group, Shanghai, China) was used for all experiments.

### 3.2. Preparation of Polyelectrolyte–Tobramycin Complexes

Due to the lack of commercial availability of HA, the acidic form of the polysaccharide was obtained from NaHA using an anionic exchange resin, as reported by Battistini et al. [24].

The ionizable carboxylic groups of both NaHA and HA were determined by using the differential scanning potentiometry technique [94], showing a value of 2.45 ± 0.2 mmol/g for HA. In the case of NaHA, the error due to the sodium led to a theoretical determination with a value near the ionizable carboxylic groups of the HA. The same values of ionizable groups were used for the acid and salt forms.

The complex HA-TOB was obtained by dispersing 0.3% *w*/*v* of the PE in deionized water and by adding the appropriate amount of TOB to neutralize 100% of the ionizable groups. Finally, the complex had the pH corrected to a value of 7.0 ± 0.5. On the other hand, the preparation of the complex NaHA-TOB was slightly different. The PE and the drug were dispersed separately, the pH was corrected to 7.0 ± 0.5 and then mixed together to avoid heterogeneous precipitation.

The spray-drying technique was used to obtain the complexes in the solid state using a spray dryer Büchi-B290 (Büchi^®^ Labortechnik, Flawil, Switzerland) according to the following parameters: an inlet temperature of 120 °C; nozzle: 0.7 mm; aspiration rate: 35 m^3^/h; an air flow rate of 600 L/h; and a feed rate of 4 mL.min^−1^. The complex in the solid state was removed from the vessel, stored for several hours under vacuum, and then the yield was measured by weighing it immediately on an analytical balance (Ohaus Adventurer^TM^, Ohaus, Parsippany, NJ, USA) according to the following equation (Equation (1)):

(1) $$Y(\%)=\frac{Ws}{Wt}\times 100$$

where *Ws* corresponds to the solid obtained after the spray-drying procedure, while *Wt* corresponds to the total amount of solid used to prepare the respective complexes.

All the powders acquired were stored in a vessel tightly sealed at 25 °C and under moisture protection. Simultaneously, a physical mixture (PM) between HA + TOB and NaHA + TOB was prepared in a mortar by mixing the same proportions as the complexes HA-TOB and NaHA-TOB, respectively. This PM was always used as a control during the physicochemical characterization of the complexes.

### 3.3. Physicochemical Characterization of HA-TOB and NaHA-TOB in Solid State

#### 3.3.1. Infrared Spectroscopy

The infrared spectra (FT-IR) of HA-TOB, NaHA-TOB, PMs of each system, and raw materials were obtained in the range of 4000 to 650 cm^−1^. An FT-IR spectrometer (Cary 630 FT-IR spectrometer, Agilent^®^, Santa Clara, CA, USA) programmed to a spectral resolution of 4 cm^−1^ and an average of 40 scans per spectrum was used. The samples were analyzed using the OMNIC 8.3 software.

#### 3.3.2. Thermal Analysis

The thermal behavior of HA-TOB, NaHA-TOB, PMs, and raw materials were performed using two techniques: differential scanning calorimetry (DSC) and thermogravimetric analysis (TGA), using the TA Discovery^®^ series instrument equipped with the Trios^®^ TA instrument program (TA Instruments, New Castle, DE, USA).

Approximately 2–4 mg of each sample was weighed and sealed in a non-airtight aluminum container. Heating was carried out at a ramp of 10 °C/min under a nitrogen atmosphere at 50 mL/min. DSC analysis was run from room temperature to decomposition temperature (200–250 °C), while TGA was performed up to a temperature of 400 °C.

#### 3.3.3. Powder X-Ray Diffraction

Powder X-ray diffraction (PXRD) was used to evaluate the crystalline or amorphous nature of HA-TOB, NaHA-TOB, PMs, and raw materials. A diffractometer (Philips PW1800, Amsterdam, The Netherlands) using Cu Kα radiation (λ = 1.5418 Å) was operated at 40 kV and 100 mA. Results were collected at an angular range of 5 to 60° using a step method, with a step of 0.02° and a scan rate of 1 s per step.

### 3.4. Powder Characterization

#### 3.4.1. Scanning Electron Microscopy

The morphologies of the powders HA-TOB, NaHA-TOB, and TOB were studied using a scanning electron microscope (FE-SEM Zeiss Σigma, Carl Zeiss AG, Oberkochen, Germany) at different magnifications. Samples were prepared by weighing about 1–2 mg of powder on a carbon ribbon pre-mounted on an aluminum support and metallized with gold/palladium. The images were captured using an electron beam accelerating voltage of 3 kV.

#### 3.4.2. Density Determination and Flow Properties

The bulk (δ_B_) and tapped (δ_T_) densities of HA-TOB and NaHA-TOB were studied. Each determination was performed in triplicate, and the density was calculated as the ratio of powder weight to the volume occupied by the powder.

For δ_B_ determination, an accurately weighed amount of complex, between 0.5 and 1 g, was placed and gently introduced into a 10 mL calibrated test tube avoiding compaction. The powder was then carefully leveled, and the volume was read to the nearest graduated unit. The δ_T_ was studied by manually compacting the powder previously introduced into the test tube until no further changes in volume were observed.

From these values, the Carr’s index (CI) and Hausner’s ratio (HR) were determined, according to the following equations:

(2) $$\mathrm{CI}(\%)=100\times \frac{\delta_{T}-\delta_{B}}{\delta_{T}}$$

(3) $$\mathrm{HR}=\frac{\delta_{T}}{\delta_{B}}$$

#### 3.4.3. Particle Size Distribution by Laser Diffraction

The geometrical particle size distribution (PSD) for HA-TOB and NaHA-TOB was carried out using a Mastersizer 3000 laser diffraction equipment, connected to the Aero S powder disperser (Malvern Panalytical, Malvern, UK). The experiments were performed at a pressure of 4.0 bar, and the average PSD of each complex was obtained from triplicate measurement.

The results are reported as volumetric diameter Dv90, Dv50, and Dv10, which represent the diameter below which 90, 50, and 10% of the population is found, respectively. Additionally, the width of the distribution or SPAN was studied and calculated according to the following equation:

(4) $$\mathrm{SPAN}=\frac{\left(Dv90-Dv10\right)}{Dv50}$$

#### 3.4.4. Moisture Content Uptake

The moisture uptake was evaluated at room temperature under two different relative humidities (RHs), 31 and 76%, in order to simulate normal and extreme conditions, respectively. For this purpose, 100 mg of HA-TOB and NaHA-TOB were weighed into centrifuge microtubes (Eppendorf^®^ tubes, Córdoba, Argentina). Three replicate samples were kept in hermetically sealed microtubes, which were placed inside a closed container kept at 25 °C and containing silica gel to achieve nearly 0% RH, and then in containers containing calcium chloride and sodium chloride saturated solutions to achieve 31 and 76% RH, respectively.

The samples were kept under these conditions until the weights of two consecutive weighing were constant. The results were expressed as the percentage increase by weight due to the uptake of moisture at different RHs.

### 3.5. In Vitro Biopharmaceutical Performance

#### 3.5.1. Analytical Quantification of TOB

TOB lacks a chromophore, preventing detection by UV-Vis spectroscopy. Consequently, it required a derivatization process to be quantified by high-performance liquid chromatography (HPLC) using a UV-Vis detector (ESC03, ECOM^®^ spol.s r.o., Prague, Czech Republic), as described in the USP37 monograph [95]. The concentration of TOB in the HA-TOB and NaHA-TOB was determined by HPLC (Shimadzu Corporation, Kyoto, Japan). An isocratic method was employed, along with a 3.9 × 300 mm with an L1 packing, Luna C18 and 10 µm. The detector was set at 365 nm and the flow rate at 1.2 mL/min, with an injection volume of 20 µL. The mobile phase was prepared by dissolving 2 g of Tris hydroxymethyl aminoethane in 800 mL of ultrapure water, and to that solution, 20 mL of 1N sulfuric acid was added and finally diluted to 2000 mL with acetonitrile. The calibration curve was performed in triplicate in a concentration range of 0.006–1.7 mg/mL.

#### 3.5.2. Aerodynamic Performance Assessment

The aerodynamic particle size distribution was analyzed by using the Next Generation Impactor (NGI, Copley Scientific Limited, Nottingham, UK), which is composed of a mouthpiece adapter in which the inhaler device will be connected, an induction port simulating the throat, the seven stages with a decreasing cut-off diameter, and, finally, the micro-orifice collector (MOC). An amount of 30 mg of powder was placed into an HPMC QUALI-V I size 3 capsule (Qualicaps, Madrid, Spain). The capsule was inserted into a RS01^®^ inhaler device (Plastiape, Lecco, Italy) and connected to the mouthpiece adapter. In order to avoid particle bouncing, 2 mL of Tween^®^ 80 1% *v*/*v* was added to each cup of the seven stages and left until evaporation. A flow rate of 65 L/min was achieved with a vacuum pump (SCP5, Copley scientific Ltd., Nottingham, UK) in order to produce a pressure drop of 4 kPa inside the inhaler and left activated for a period of 4 s. The amount of TOB deposited at the different parts of the NGI was measured by HPLC. Each experiment was performed in triplicate, and the results were expressed as the mean with its standard deviation (SD).

This analytical quantification allowed the analysis of different dry powder formulation parameters according to USP specifications [74]. The emitted dose (ED) is defined as the amount of the delivered dose from the induction port to the MOC at any aerodynamic diameter, while the emitted fraction (EF) is the ratio between the ED and the total amount of powder present in the capsule at the beginning of the experiment. The fine particle fraction (FPF) and the extra fine particle fraction (ExtraFPF) are defined as the inhalable powder fractions with an aerodynamic diameter of less than 5 μm and 3 μm, respectively. These fractions are calculated as the ratio of the mass of particles with a diameter of less than 5 or 3 μm to the ED. Finally, the mass median aerodynamic diameter (MMAD) is defined as the aerodynamic size, which possesses 50% of the powder population, and its determination is realized by plotting the cumulative percentage of mass (from stage 1 to MOC) versus the cut-off diameter on a logarithmic scale.

#### 3.5.3. Dissolution Study

Dissolution studies were carried out using a modified version of the bicompartmental Franz cells in which a cellulose filter (Whatman^®^ qualitative filter paper, Grade 1, Cytiva, Marlborough, MA, USA) was placed between the donor and the receptor compartments. The powder was sieved with a 560–630 μm sieve prior to the test. An amount of powder containing approximately 7 mg of TOB was placed carefully on the donor compartment above the filter, avoiding compaction or agglomeration. The test was performed at 37 ± 0.1 °C under gentle constant stirring and a dissolution media of phosphate-buffered solution (PBS) with pH 7.4 was used. The receptor compartment was filled with approximately 16 mL of the dissolution media, and 1 mL was added to the donor compartment to ensure a complete wetting of the powder. Samples were collected at predetermined time points of 15, 30, 60, 90, and 120 min and were replaced with the exact volume of fresh thermostated medium. The amount of TOB dissolved was quantified by HPLC. All the experiments were carried out in triplicate, and sink conditions were maintained. The results were expressed as the mean with their SD.

### 3.6. Microbiological Activity Assays

#### 3.6.1. Inoculum Preparation

The inoculum standardization was realized from *S. aureus* ATCC 29213 and *P. aeruginosa* ATCC 27853 cultures left overnight in a tryptone soy agar (TSA, Britania, Buenos Aires, Argentina) by suspending bacterial colonies in a Mueller Hinton broth (MHB, Britania, Argentina). The bacterial suspension was diluted until reaching a 0.08–0.1 absorbance measured at 600 nm employing UV-Vis spectrophotometry (Evolution 300, Thermo Electron Corporation, Waltham, MA, USA), which corresponds to 0.5 of the MacFarland scale and a density value of 1.5·10^8^ CFU/mL, approximately.

#### 3.6.2. Minimum Inhibitory Concentration (MIC) and Minimum Bactericidal Concentration (MBC) Determination

The minimum inhibitory concentration (MIC) and the minimum bactericidal concentration (MBC) of the TOB, HA-TOB, and NaHA-TOB were determined using *P. aeruginosa* strain ATCC 27853 and *S. aureus* strain ATCC 29213. The MIC and MBC against both strains previously mentioned were determined with the microplate dilution method, according to Clinical and Laboratory Standards Institute (CLSI) standards [96]. Samples of 100 μL of the bacterial inoculum suspension as well as the MHB were incorporated into each well of a 96-well plate. Serial dilutions (64–0.5 μg/mL) from the antimicrobial samples were performed and added to the microplate in a volume of 100 μL and then incubated at 37 °C for 24 h. The establishment of the MIC was realized by determining the lowest concentration at which the presence of turbidity was not noticeable to the naked eye, corresponding to the inhibition of the bacterial growth. This concentration and two below were plated on Mueller Hinton agar (MHA) and incubated for 24 h at 37 °C to determine the MBC, which is considered the lowest antimicrobial concentration in which the initial inoculum is reduced by 99.9%.

#### 3.6.3. Biofilm Inhibition and Eradication Studies

The inhibition and eradication assays were performed using *P. aeruginosa* strain ATCC 27853 (1.5·10^8^ CFU/mL) and *S. aureus* strain ATCC 29213 (1.5·10^8^ CFU/mL), and the crystal violet staining method was employed. The samples studied were TOB, HA-TOB, and NaHA-TOB, which were dissolved in sterile water at a concentration of 500 or 1000 times higher than their MBC values for the inhibition and eradication studies, respectively. Serial dilutions (factor 2) were made from the samples. In the case of the inhibition study, 100 μL of each sample dilution was placed in a 96-well microplate, containing the same volume of the inoculum (*P. aeruginosa* or *S. aureus*), and incubated at 37 °C for 24 h, then the supernatant was discharged, and the microplate was washed with PBS pH 7.4 buffer. In parallel, to study the eradication of the biofilm, 200 μL of the respective inoculum was placed in a 96-well microplate and incubated at 37 °C for 24 h after which the supernatant was discharged and replaced with 200 μL of each sample dilution, followed by a second incubation at 37 °C for 24 h. The biofilm present in both cases, i.e., inhibition and eradication studies, was stained with crystal violet 0.1% *w*/*v* for 20 min, and afterwards, the supernatant present in the microplate was washed with PBS pH 7.4 buffer. The crystal violet present in the biofilm and/or bacteria was extracted using ethanol and then quantified by UV-Vis spectroscopy at 595 nm (Synergy HT, BioTek Instruments, Winooski, VT, USA). Each experiment was performed in triplicate.

We obtained SEM images of the inhibition and eradication of the biofilm produced by both strains according to the methodology previously described.

### 3.7. Statistical Analysis

The statistical analysis was performed using Prism 9.0 software (GraphPad, Boston, MA, USA). The one-way ANOVA test was employed to evaluate the statistical significance, considering p values lower than 0.05 as indicative of statistical difference.

Kinetics studies of dissolution from the PE were evaluated with the similarity factor, *f*_2_, as presented in Equation (5):

(5) $$f_{2}=50\cdot log\left\{(1+\left(\frac{1}{n}\right)\sum_{t=1}^{n}\;(R_{t}-P_{t}{)}^{2}{)}^{-0.5}\cdot 100\right\}$$

where *n* is the number of sampling points, and *R_t_* and *P_t_* are the cumulative percentages of the dissolved drug at each time, *t*. Two profiles are considered different when the value of *f*_2_ calculated between them is lower than 50.

## 4. Conclusions

Dry powders based on TOB and HA or its salt, NaHA, as ionic complexes were successfully achieved by employing simple and scale-up techniques. The ionic interactions between the mentioned precursors were confirmed by FT-IR, PXRD, and thermal analysis, although the presence of non-interacting TOB in the NaHA-TOB complex was observed. Bulk and tapped densities, the morphology and size of the powders showed that both complexes are suitable for reaching the deep lungs due to their low densities, micronized size, and spherical shape, which decreases the likelihood of lung irritation. Additionally, the aerodynamic performance of the complexes revealed that they are good candidates for administration by pulmonary route. The dissolution performance of HA-TOB and NaHA-TOB showed a rapid dissolution that can possibly favor the maintenance of the powders in the site of action, avoiding the local defenses. Finally, microbiological studies showed that both complexes did not affect the antimicrobial properties against the *P. aeruginosa* and *S. aureus* of TOB despite the ionic PE-D interaction, as well as good antibiofilm activity even improving it, especially for the HA-TOB complex. These interesting properties of the complexes highlighted the potential benefits in the treatment of pulmonary infections in patients with CF.

## Figures and Tables

**Table 1:** Bulk and tapped density, Carr index and Hausner’s ratio of the HA-TOB and NaHA-TOB.

Sample	δ_bulk_ (g/cm^3^)	δ_tapped_ (g/cm^3^)	Carr Index	Hausner’s Ratio
HA-TOB	0.20 ± 0.05	0.4 ± 0.1	40 ± 4	1.7 ± 0.1
NaHA-TOB	0.15 ± 0.03	0.22 ± 0.03	34 ± 5	1.5 ± 0.1

**Table 2:** Particle size distribution expressed as volumetric diameter and distribution width (SPAN).

Samples	Dv10 (µm)	Dv50 (µm)	Dv90 (µm)	SPAN
HA-TOB	1.14 ± 0.02	2.90 ± 0.02	6.37 ± 0.07	1.70 ± 0.05
NaHA-TOB	1.48 ± 0.03	4.14 ± 0.01	9.80 ± 0.02	2.01 ± 0.07

**Table 3:** Aerodynamics parameters of HA-TOB and NaHA-TOB obtained by NGI.

Sample	EF (%)	FPF (%)	ExtraFPF (%)	MMAD (µm)
NaHA-TOB	82 ± 1	60 ± 2	25 ± 1	2.5 ± 0.01
HA-TOB	78.9 ± 0.7	52 ± 3	20 ± 2	2.6 ± 0.03

EF: emitted fraction; FPF: fine particle fraction; ExtraFPF: extra fine particle fraction; MMDA: mass median aerodynamic diameter.

**Table 4:** Minimum inhibitory concentration (MIC) and minimum bactericidal concentration (MBC) obtained for TOB, HA-TOB, and NaHA-TOB against *P. aeruginosa* and *S. aureus* ATCC strains.

Samples	*P. aeruginosa*		*S. aureus*	
	MIC (µg/mL)	MBC (µg/mL)	MIC (µg/mL)	MBC (µg/mL)
TOB	1	2	0.5	1
HA-TOB	0.5	1	0.25	0.5
NaHA-TOB	0.25	0.25	0.125	0.25

## Data Availability

Data are contained within the article.
